# What Killed Him?

**Published:** 1870-08

**Authors:** 


### HALL'S

### JOURNAL OF HEALTH.

VOL. XVII. — AUGUST, 1870. — No. VIII.

### WHAT KILLED HIM?

WHEN the news was read to us from the *New York Evening Mail*, that Charles Dickens was dead, the instant mental inquiry was, "What killed him?" A man does not die naturally at fifty-eight. Everybody calculated that he would interest and delight the world with his writings for a good many years to come; and yet his light has gone out in the night of the grave so soon; too soon for the world's good. He put that light out himself, by dining and wining. He drank largely, and ate sumptuously, else he might have lived, the world's delight, for a good dozen years to come. The papers contain all sorts of trashy moralizing about his having written too much, and applied his brain too much; and nine persons out of ten are under some kind of impression that mental application shortened his life. Charles Dickens was a wine-bibber and a gourmand; has been all his days, and continued to be up to the latest moment of intelligent life; he was taken away from his seat at the festive board and carried senseless to his bed to die with the day. Leaving out the subject of preparation for the great future, we are heartily glad that he was spared the sufferings of a long, tedious and painful last illness. If Mr. Dickens had lived a temperate life, he would have lived in health a score of years to come, because he had a good constitution.

The living great should always bear in mind that the truth of history demands a faithful portraiture after they have left the stage of life. Let

THE HISTORY OF A DAY

at Gad's Hill, the 29th of July, twelve months before he died, tell the sad story. On the arrival of some friends at his house to spend the day, at half past twelve o'clock, they "commenced" with the "cider cup." Quite as innocent a name as is common to the groggeries of New York city, called "The Gem," "The Library," "The Bank," "The Office," "The Dew Drop," "The Pearl," "The Counting House," "The Sample Room." The Prince of darkness often delights to practice his calling not only in the guise of an angel of light, but in the name also. The "cider cup" was evidently a favorite drink; it had been prepared in advance; it was made of soda-water, sherry wine, brandy, lemon-peel, sugar, ice; and an herb to flavor it, called burridge, all "judiciously mixed." This was at half past twelve. Lunch came on at one o'clock; this was "completed" by a liquor which Mr. Dickens said was "peculiar to the house." After playing "Aunt Sally," and rolling balls on the grass for an hour or two, they had an "interval" at half past three for "cool brandy and water." At half past six, dinner came on with wines "irreproachable;" then came billiards, music, the drawing-room, until eleven, when "hot and rebellious liquors" wound up, in the course of an hour, the history of a day at Gad's Hill, the house of Charles Dickens. Here in the hottest of the summer days there were five deliberate "set-to's" at drink in the course of twelve hours. This may not have been the history of every day with the world's favorite, but no man arrives at a drinking ability of that style, without a great multitude of rehearsals preceding it. This is written in no unlovely feeling towards Mr. Dickens, but as a warning to the living, as good and great as he, and as useful, that they come not into the same condemnation.

There is a great practical wrong done to humanity in attributing any ill result to the wrong cause; for while attempting to avoid that wrong cause, the real one is permitted to remain in full force, accomplishing its sad havocs all the time without restraint. It ought to be known as a world-wide truth, that, of itself,

HARD STUDY KILLS NOBODY.

Thought is the life of the brain, as exercise is the life of the body. There can be no more such a thing as a healthy brain, as to the mental department, without thought, study, than there can be a healthful body without exercise. And as physical exercise preserves the body in health, so thought, which is the exercise of the brain, keeps it well. But here the parallel ends; we may exercise, work too much, but we cannot think too much, in the way of expressing ourselves, for both writing and talking are a relief to the mind; they are in a sense its play; its diversion. Pent up thought may kill, as pent up steam wrecks the locomotive. The expression of thought, is like working off the steam from the boiler. When clergymen break down, or public men, or professors in colleges, or other literary institutions, get sick and die, the universal cry is, "over-study," "too much responsibility," "too much mental application." It is never so; not in a single case since the world began; we defy proof, and will open our pages to any authenticated case. If a man will give himself sleep enough, and will eat enough nutritious food at proper intervals, and will spend two or three hours in the open air every day, he may study, and work and write, until he is as gray as a thousand rats, and will be still young in mental vigor and clearness. Where is the man of renown who lived plainly, regularly, temperately, and died early?

It does not answer in an argument of this sort, which practically interests every scholar in Christendom, to kick up a dust or make a great hullaballoo, and in this noise and dust, run away exclaiming, "It is all nonsense, many a hard student has died prematurely, as a consequence of too intense application;" give us the name of one.

We are really angry at Charles Dickens's virtual suicide, because his is a world's loss, of a far higher pleasure than the wine cup, or the feast of fat things ever gave. We never read one of his novels, and never expect to; to spend a whole day, or the leisure of a dozen days, in reading a book, and then, when you close it, have the thought flit across the mind, "It's all a fabrication," is perfectly intolerable; don't know what it might be on a rainy day, in a country inn, or shut up on shipboard; but in grand old New York, where a fellow can walk up and down Broadway and stop whenever he has a mind to, to look in at the shop windows or run over the titles of books on the rich shelves of Hurd & Houghton in Astor Place, or take a "fit" at Baldwin the clothier, or have a drive in Central Park and look at all the pretty girls; why, we had a great deal rather do any of these things, or take a run up to Cornwall Heights in the *Mary Powel*, of a summer afternoon, or on an Albany boat in the balmy fresh morning air, than to read any novel ever published. We have no taste for imagination, but anything we can see or feel or hear and make use of, or turn to some practical account, why, bring it along.

Mr. Dickens never wrote a single line, friendly to the Christian religion; so we gather from what the papers have said of him since his death; has never advocated practical temperance, but has commended wine drinking; and has made a butt of clergymen, and to that extent has been "against" HIM, who died to save. At the same time, he has so largely coöperated with the work of the Church in drawing charitable attention to the poor, more and more efficiently than all the writers of fiction together, who have ever lived, that he must be considered one of the greatest benefactors of the age; and whether designedly or not, for the motive must be left to the beneficent judgment of the All Seeing Eye, he has been a world's missionary; he has been a John the Baptist, a clearer of the way for the coming of a more blessed Evangel. In no period of the world's history, not in all the world's history up to the year eighteen hundred, has the one hundreth part of the attention of men been given to the care and elevation of the poor and the degraded, as within the last thirty years; that the writings of Charles Dickens have directly contributed to this result, and have largely so contributed, it is impossible for any intelligent man to deny. In so doing, he worked hard, worked long, worked enthusiastically, and we humbly trust, that opposite his name in the book of life it may be written,

"HE DID WHAT HE COULD,"

considering the opportunities he had of religious education in early life. Reader, have you done as much for humanity, with all your greater light from a religious training, from a mother's prayers and teachings, from Sunday-school facilities and the influences of Christian ministers, from infancy to manhood? Mr. Dickens had almost none of these, hence his name may be higher on the list than yours or mine.

### DICKENS'S LITERARY HABITS.

"He took breakfast at eight, answered all the letters received that day, wrote until one, had a lunch, walked twelve miles as a matter of course every day, dined at six and spent the remainder of the day with his family, entertaining his friends." This was the general habit of his life, at least of late years, by which it appears that his literary labors did not exceed four hours in twenty-four, and then to talk about his premature death as a result of excessive mental labor, is the most absurd of all absurd figments of the imagination.

